# Integrated strategy for in vitro characterization of a bileaflet mechanical aortic valve

**DOI:** 10.1186/s12938-017-0314-2

**Published:** 2017-02-16

**Authors:** Francesca Maria Susin, Stefania Espa, Riccardo Toninato, Stefania Fortini, Giorgio Querzoli

**Affiliations:** 1 Department of Civil and Environmental Engineering, Sapienza University of Rome, Rome, Italy; 2 Cardiovascular Fluid Dynamics Laboratory HER, Department of Civil, Environmental and Architectural Engineering, University of Padua, Padua, Italy; 3 Department of Civil, Environmental Engineering and Architecture, University of Cagliari, Cagliari, Italy

**Keywords:** Pulse duplicator, Image velocimetry, Valve leaflets dynamics, Haemolysis index

## Abstract

**Background:**

Haemodynamic performance of heart valve prosthesis can be defined as its ability to fully open and completely close during the cardiac cycle, neither overloading heart work nor damaging blood particles when passing through the valve. In this perspective, global and local flow parameters, valve dynamics and blood damage safety of the prosthesis, as well as their mutual interactions, have all to be accounted for when assessing the device functionality. Even though all these issues have been and continue to be widely investigated, they are not usually studied through an integrated approach yet, i.e. by analyzing them simultaneously and highlighting their connections.

**Results:**

An in vitro test campaign of flow through a bileaflet mechanical heart valve (Sorin Slimline 25 mm) was performed in a suitably arranged pulsatile mock loop able to reproduce human systemic pressure and flow curves. The valve was placed in an elastic, transparent, and anatomically accurate model of healthy aorta, and tested under several pulsatile flow conditions. Global and local hydrodynamics measurements and leaflet dynamics were analysed focusing on correlations between flow characteristics and valve motion. The haemolysis index due to the valve was estimated according to a literature power law model and related to hydrodynamic conditions, and a correlation between the spatial distribution of experimental shear stress and pannus/thrombotic deposits on mechanical valves was suggested. As main and general result, this study validates the potential of the integrated strategy for performance assessment of any prosthetic valve thanks to its capability of highlighting the complex interaction between the different physical mechanisms that govern transvalvular haemodynamics.

**Conclusions:**

We have defined an in vitro procedure for a comprehensive analysis of aortic valve prosthesis performance; the rationale for this study was the belief that a proper and overall characterization of the device should be based on the simultaneous measurement of all different quantities of interest for haemodynamic performance and the analysis of their mutual interactions.

**Electronic supplementary material:**

The online version of this article (doi:10.1186/s12938-017-0314-2) contains supplementary material, which is available to authorized users.

## Background

Incidence of heart valve diseases is growing in western countries with population age and life expectancy increasing [1, 2]. Satisfactory transvalvular haemodynamic conditions and heart pump function are usually restored at the short- and mid-term after valve replacement. Nevertheless, current prostheses are still quite far from representing the ‘optimum prosthetic valve’. Mechanical heart valves (MHVs) express high durability but induce flow patterns different from those observed in healthy subjects [3, 4]. Also, MHVs studies highlighted a sharp tendency to thrombus formation, which requires life-long anticoagulant therapy [2], as well as to haemolysis [5]. On the other hand, biological prostheses haemodynamics is usually nearly physiological but they show short durability mainly due to leaflets stiffening caused by shear stresses and calcification phenomena [6–8]. In both cases the fluid–structure interaction plays a fundamental role in determining prosthesis functionality, hence a thorough analysis of flow characteristics close to the valve is essential to assess its overall performance [9]. The work by Dasi et al. [10], who described the interaction between vorticity and leaflet kinematics of a bileaflet mechanical heart valve (BMHV), is a first important step in that direction. However, literature usually focuses on either global functionality, to assess whether the artificial valve overloads heart work, or local functionality, to quantify the shear stress field and its potential effects in terms of blood cells damage and leaflets degeneration. Several in vitro and in vivo studies were aimed at the experimental estimation of global haemodynamic parameters as the transvalvular pressure drop, the effective orifice area (EOA) or the regurgitant and leakage volumes (see e.g. [11–16]). As for valve dynamics, attention has been most devoted to study the behavior in time of the valve area for both biological and mechanical prosthesis [17–20], while the leaflets motion of bileaflet mechanical heart valve (BMHV) has been somehow less investigated despite the importance of the issue [10, 21–23]. Several numerical studies focused on the occluders dynamics using fluid–structure interactions approach [22, 24–27]. Flow patterns and shear stress distribution in correspondence of the valve have been extensively investigated both numerically [6, 24, 28, 29] and in vitro [20, 30–34]. Moreover, several literature works deal with red blood cells (RBCs) or platelets damage, providing haemolysis laws to characterize the dangerousness of the flow through the prosthetic device [35–39] or of the valve itself [40].

Even though these studies provide a solid and recognized base as single interpretation of a complex phenomenon, a unique strategy to characterize the valve overall hydrodynamic performance is still vacant. To this aim, this study proposes an integrated approach able to provide simultaneous in vitro measurements of (1) pressure and flow waves across a prosthetic valve; (2) leaflets position in time; (3) flow field and shear stress distribution (near and far fields) downstream of the valve (notice that all these quantities are required by international standards), and to highlight mutual interactions between all investigated mechanisms. The tests were performed in a mock loop simulating the human systemic circulation in a model of healthy ascending aorta.

## Methods

**Fig. 1:**
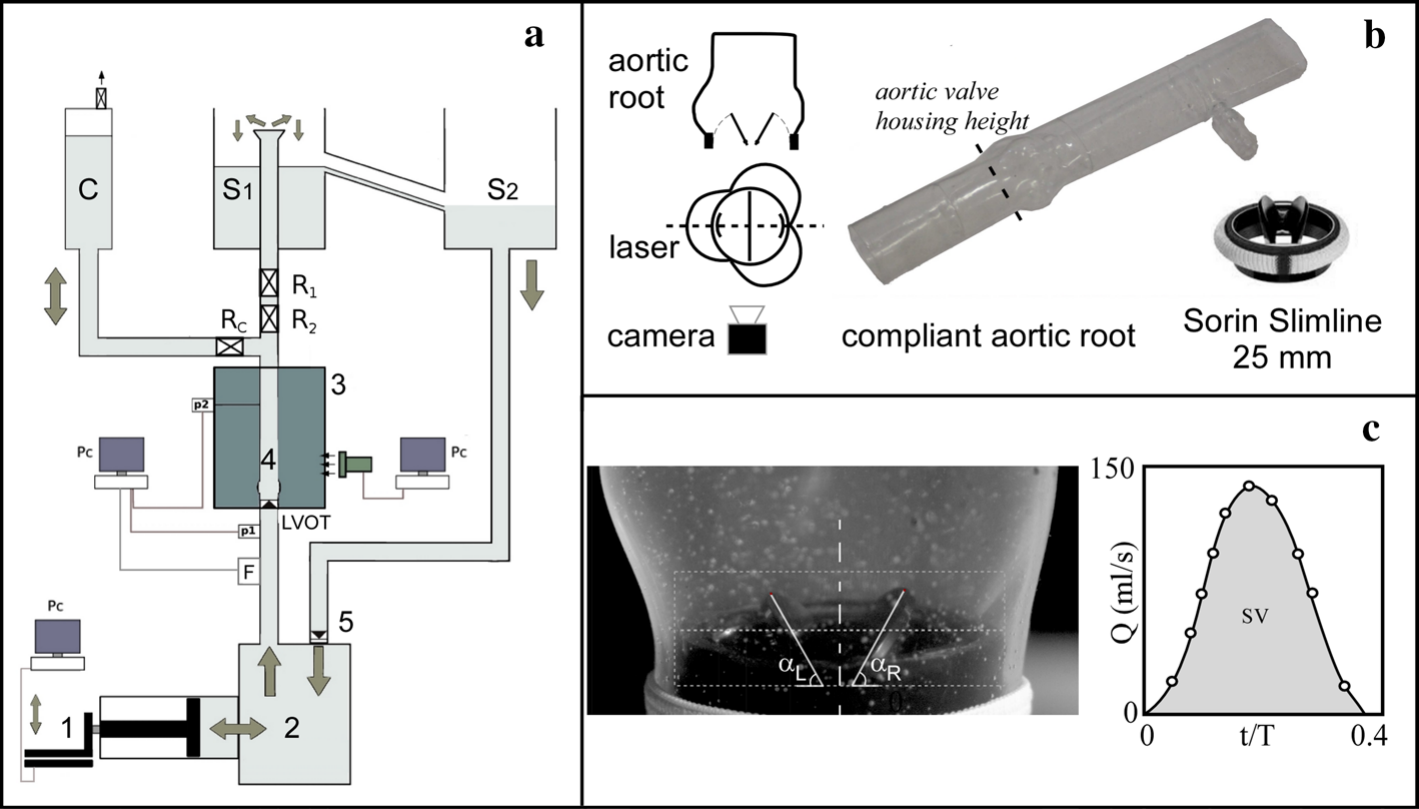
**a** Sketch of the experimental apparatus: *1* Piston pump; *2* ventricular chamber; *3* aortic chamber; *4* aorta; *5* mitral valve; *R1* and *R2* peripheral resistance; *RC* compliance flow regulator; *C* compliance chamber; *S1* right atrial chamber, *S2* left atrial chamber. **b** Set up of camera, laser sheet, valve and aortic root mutual position; aortic root model plus the adopted mechanical valve. **c** Measuring tool for leaflet tilting angles [*right* (α_R_) and *left* (α_L_)], and chosen time instants for leaflets dynamic measurements, in the ejection phase. The *grey area* represents the SV pumped into the aorta

The apparatus here adopted is the pulse duplicator (PD) that was already described in its basic functional elements and capability of reproducing physiological flows [41–47]. The PD has been adapted with an ad-hoc simplified replica of the human ascending aorta (AA) connected to the left ventricle outflow tract (LVOT) (Fig. 1a). AA was made of transparent compliant silicone rubber (Sylgard-184, Tensile Modulus 1050 psi and 2 mm thickness) by dipping technique, choosing shape and dimensions in accordance to average adult population characteristics, sinuses of Valsalva included (aortic annulus inner diameter D = 25 mm, AA height H = 70 mm, aortic root radius/aortic radius = 1.4, height of sinuses of Valsalva = 20 mm). As discussed in detail in [46] and in [47], the distensibility of the aorta in the interval between the systolic peak and the diastole, has been reproduced by imposing a correct percentage diameter change (10–16%) during the cardiac cycle accordingly to the physiological range [48, 49]. A bileaflet Sorin Bicarbon Slimline valve [50, 51] (nominal diameter d_v_ = 25 mm, comprehensive of the suture annulus—Fig. 1b) commonly used for replacement was placed at surgical height inside the aortic root, using a proper housing. Valve-mock root mutual position provides a typical orientation [30], with a leaflet dedicated to one sinus and the other in correspondence to a commissure (Fig. 1b).

Two piezoelectric sensors (PCB Piezotronics^®^ 1500 series, Fig. 1a -P_1_ and P_2_-) located respectively 3,5D upstream and 6,25D downstream the aortic valve, provided aortic (p_a_) and ventricular (p_v_) pressure. An electromagnetic flowmeter (501D Carolina Medical Electronics, Fig. 1a -F-) recorded the aortic flow rate during cardiac cycle. An example of recorded forward flow rate Q in non-dimensional time t/T, where T is the dimensional period of the cycle, is reported in Fig. 1c. Positive Q gives the systolic outflow rate while the grey area equals the ejected stroke volume (SV). The time law of the ventricle volume change was assigned to mimic a physiological behavior (the flow curve used in the commercial, FDA approved, ViVitro^®^ mock loop system). To fulfill the geometric similarity a geometric aspect-ratio 1:1 was set on the investigated area. Farther, since water (whose viscosity is about one-third of that of the blood) was used as working fluid, to respect the dynamic similarity, for a given physiological SV, the period of the cardiac cycle adopted in the experiments was set equal to three times the physiologic one. In the considered settings of the flow control parameters the peak velocity varied in the range 0.15–0.25 m/s and non-dimensional parameters, Reynolds and Womersley numbers, resulted respectively 2500 < Re < 4500 and 14 < Wo < 17. The similarity with respect to the leaflet motion is also matched since scale effects are not expected [43].

### Pressure and EOA measurements

The ability of the PD to accurately reproduce physiological ventricular and aortic pressures was assessed by comparing experimental and real pressure behaviors in both shape and reference values (min and max systolic pressures and mean aortic pressure $\overline{{{\text{p}}_{\text{a}} }}$ over the period T). Sensitivity of the PD to haemodynamic input conditions as SV and T was also verified. To this aim we examined the variability of both the mean (evaluated over the period of forward flow) transvalvular pressure drop $\Delta {\text{p}}_{\text{m}} = \overline{{\left( {{\text{p}}_{\text{v}} - {\text{p}}_{\text{a}} } \right)}}$ and the EOA corresponding to five different combinations of the parameters SV and T, listed in Table 1.

**Table 1:** Experimental parameters

Test	SV (ml)	T (s)	Equivalent beat rate (bpm)	Δp_m_ (mm_Hg_)	Q_rms_ (l/min)	EOA (cm^2^)
1	64	1.8	100	22.23	6.53	1.34
2	54	2.4	75	13.23	4.39	1.17
3	64	2.4	75	15.03	5.29	1.33
4	80	2.4	75	19.26	6.68	1.48
5	64	3.0	60	9.36	4.07	1.29

An Additional file 1 containing the pressure fields across the valve is included [see pressure_data.xls].

Haemodynamic input conditions SV and T adopted in PD sensitivity analysis tests. Fundamental global haemodynamic parameters calculated as averages over 100 non-consecutive cycles are also reported; Δp_m_: mean transvalvular pressure drop over the ejection period; Q_rms_: root mean square aortic flow rate over the ejection period; EOA. Recall that to ensure dynamic similarity between the in vitro model and the real environment, experimental flow rate was set to 1/3 of the physiological one.

It has to be noted that Δp_m_ and the EOA are the global parameters that have to be checked in vitro to assess the systolic haemodynamic performance of implanted heart valves according to the European Standard EN ISO 5840 [52]. In particular, the EOA has to be calculated as:

1 $$ {\text{EOA}} = \frac{{{\text{Q}}_{\text{rms}} }}{{51.6\sqrt {\frac{{\Delta {\text{p}}_{\text{m}} }}{\uprho}} }} $$

where Q_rms_ is the flow root mean square in the ejection period measured in ml/s and ρ is the fluid density in g/cm^3^, thus resulting in EOA given in cm^2^ when Δp_m_ is in mmHg.

### Haemolysis index

To estimate blood cell damage due to mechanical stress, usually the haemolysis index (HI), is considered. HI(%) is defined as the ratio between the increase in plasma free haemoglobin (∆H_b_) and the whole haemoglobin contained in a sample of blood (H_b_) exposed to the action of flow shear stress [53]. Among the proposed formulations (for a comprehensive review see [37, 53, 54]), and with the only aim of having a preliminary quantification of potential haemolysis, we adopted the power law model proposed by Giersiepen [55] used for calculating the HI for one single passage through mechanical heart valves:

2 $$ {\text{HI}}(\% ) = \frac{{\Delta H_{b} }}{{H_{b} }}100 = 3.62 \cdot 10^{ - 5} \cdot {\text{t}}_{\exp }^{0.785} \cdot\uptau^{2.416} $$

where, t_exp_ is the duration of the exposure to the ‘active’ shear stress τ.

### Leaflets dynamics

Leaflets dynamics was investigated through a semi-automatic image analysis technique. Pictures of aortic longitudinal mid-plane perpendicular to leaflets pivots were acquired by a high speed camera (Mikrotron Eosens MC1362) with spatial resolution 1280 × 1024 pixels and at 500 fps placed at an angle of 30° with respect to the valvular ring plane. Angles α_L_ and α_R_ between the valve ring plane and leaflets were measured, assuming each occluder as a line going from the leaflet top to the hinge (Fig. 1c, left). Ten instants in the ejection period were chosen as relevant to sample the tilting angles (Fig. 1c, right).

### Velocity measurements

The local flow field downstream the aortic valve between the valve ring and up about 2 cm over the sinotubular junction was measured using image analysis. To this aim, the working fluid was seeded with passive buoyant hollow glass particles (VESTOSINT 2157, D_mean_ = 30 µm, density 1.016 g/cm^3^). The symmetrical vertical mid-plane of AA was lit by a 12 W infrared laser and flow images were acquired using a Mikrotron high speed camera at 500 fps (time resolution Δt = 2 ms). Velocity fields were obtained using the Feature Tracking (FT) technique [41], in this case we considered 50 × 51 grid points, corresponding to a spatial resolution Δs = 0.78 mm. All the derived quantities needed to investigate the flow features (velocity gradients, mean flow and velocity fluctuations) were then evaluated. In particular, the maximum viscous shear stress τ_tmax_ was here calculated as [41, 56]:

3 $$ \uptau_{\rm{max} } =  \frac{{\left( {\uptau_{1 - }\uptau_{2} } \right)}}{2} = 2\upmu\left( {\text{e}_{1} - \text{e}_{2} } \right) $$

where τ_i_ and e_i_ are the eigenvalues of the stress tensor and the strain velocity tensor, respectively and μ is test fluid dynamic viscosity. Spatio-temporal resolution (Δs/D = 3 × 10^−2^; Δt/T = O(10^−3^)) was estimated high enough to identify vortex structures in the investigated region, and to follow their evolution during the cardiac cycle. Experiments were performed in four combinations of the haemodynamic input conditions, namely SV = 64 and 80 ml, and T = 2.4 and 2.6 s. For each parameter combination, 100 consecutive cardiac cycles were acquired to compute phase averaged quantities. An Additional file 2: movie file shows the trajectories reconstruction procedure in one of the performed experiments [see Tracking.avi] and the phase averaged velocity fields are also included as Additional file 3 (see “Availability of data and materials” section).

## Results

### Global flow characteristics and prosthetic valve haemodynamic performance

**Fig. 2:**
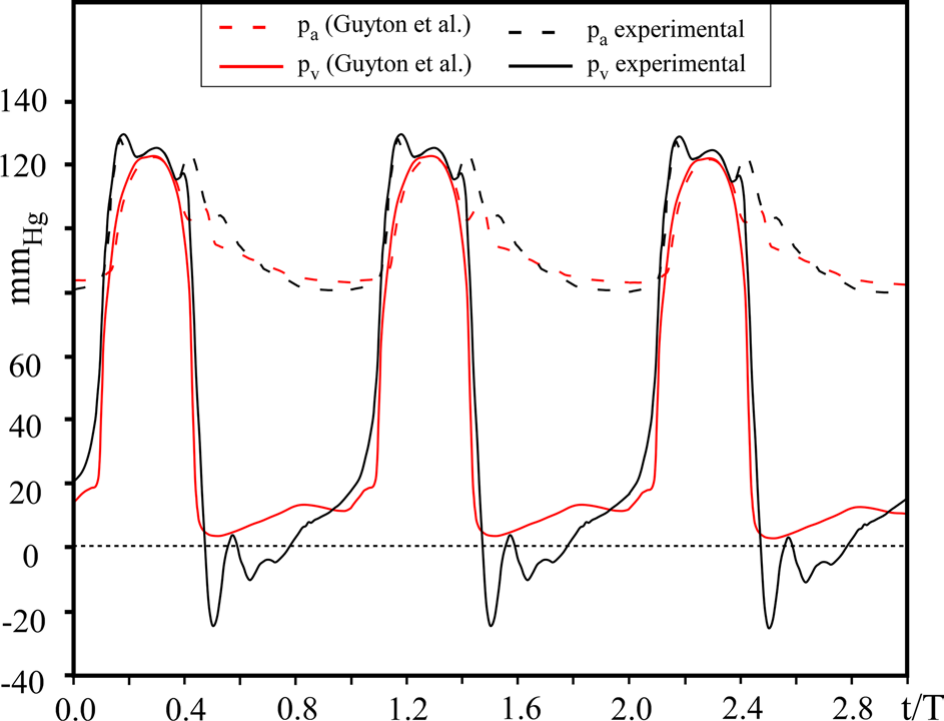
Comparison between the ventricular (p_v_) and the aortic (p_a_) pressure behavior from medical literature (*red lines*, [53]) and in vitro test with the mock loop (*black lines*)

**Fig. 3:**
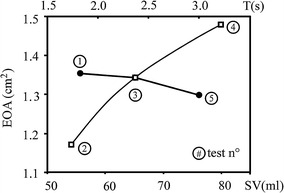
EOA as a function of the SV (*white squares*) for the fixed physiological T = 2.4 s, and as a function of the period (*black dots*), for SV = 64 ml (experiments numbered as reported in Table 1)

Physiological [57] and in vitro waveforms of ventricular and aortic pressures are compared in Fig. 2. The obtained experimental waves mimic the main physiological characteristics, including the presence of the dicrotic notch at valve closure. The presence of pressures crossing, in the forward flow phase, confirms the in vitro phenomena for the BMHVs known as leaflet fluttering, also noticed by [30]. Moreover, in vitro minimum, maximum and mean values of both p_a_ and p_v_ are in the typical physiological range (Fig. 2). These results, together with the experimental aortic forward flow wave shown in Fig. 1c, assure that our laboratory facility satisfactorily reproduces the physiological flow conditions. Also we considered the measurement of the mean transvalvular pressure drop, ∆p_m_, and the EOA as they represent the global flow parameters in the ejection phase. We tested the haemodynamic performance of the valve under the physiological pulsatile flow conditions listed in Table 1. As expected, results show that different working conditions induce different Δp_m_ and EOA values. In agreement with literature [11, 58, 59] we found that the EOA is a growing function of SV while it decreases with T (Fig. 3).

### Leaflets dynamics

**Fig. 4:**
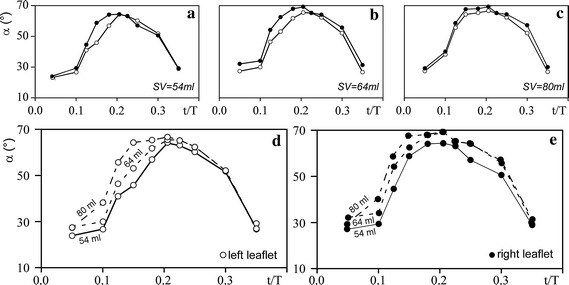
*Left* (α_L_, *white dot*) and *right* (α_R_, *black dot*) leaflet tilting angles behavior in non-dimensional time t/T. **a**–**c** show the case SV = 54, 64 and 80 ml, respectively. **d**, **e** show the trend between the same leaflet but at different SV. T = 2.4 s was used for all results

Figure 4 shows the behavior of the measured right and left leaflets tilting angles (α_R_ and α_L_, respectively) versus the non-dimensional time t/T for the three hydrodynamic conditions T = 2.4 s, SV = 54, 64 and 80 ml. The performed measurements allow to describe the movement of the two single leaflets and to highlight the possible dependence of opening and closing valve dynamics on the local and global flow characteristics. Panels a–c illustrate the asynchronous dynamics of the two leaflets, in particular during the opening phase, and show that the right leaflet usually opens at larger angle. Differences are reduced as the SV increases. Panels d and e further clarify the effect of the SV on the leaflets dynamics: during the opening phase the tilting angle increases as the SV increases, on the contrary during the closing phase the variation of the SV has a less impact on it. A possible explanation for the observed asymmetry in leaflets movement might be in even minor differences in leaflets design/construction parameters as suggested by [10], who first observed the asymmetric kinematics of BHMVs leaflets. In the present case, asymmetry might be also related to the different orientation of the two leaflets with respect to the sinuses of Valsalva, as shown by numerical predictions reported in [60]. As recently demonstrated by [61], in fact, prosthetic valve-aortic root mutual configuration strongly affects flow characteristics in proximity of the valve. Hence, it can be here speculated that the geometric mismatch between the BHMV (which has a 120° symmetry) and the root (with its 180° symmetry) implies asymmetric flow field characteristics, which in turn drive the asymmetric behavior of the two leaflets [10].

### Local transvalvular flow

**Fig. 5:**
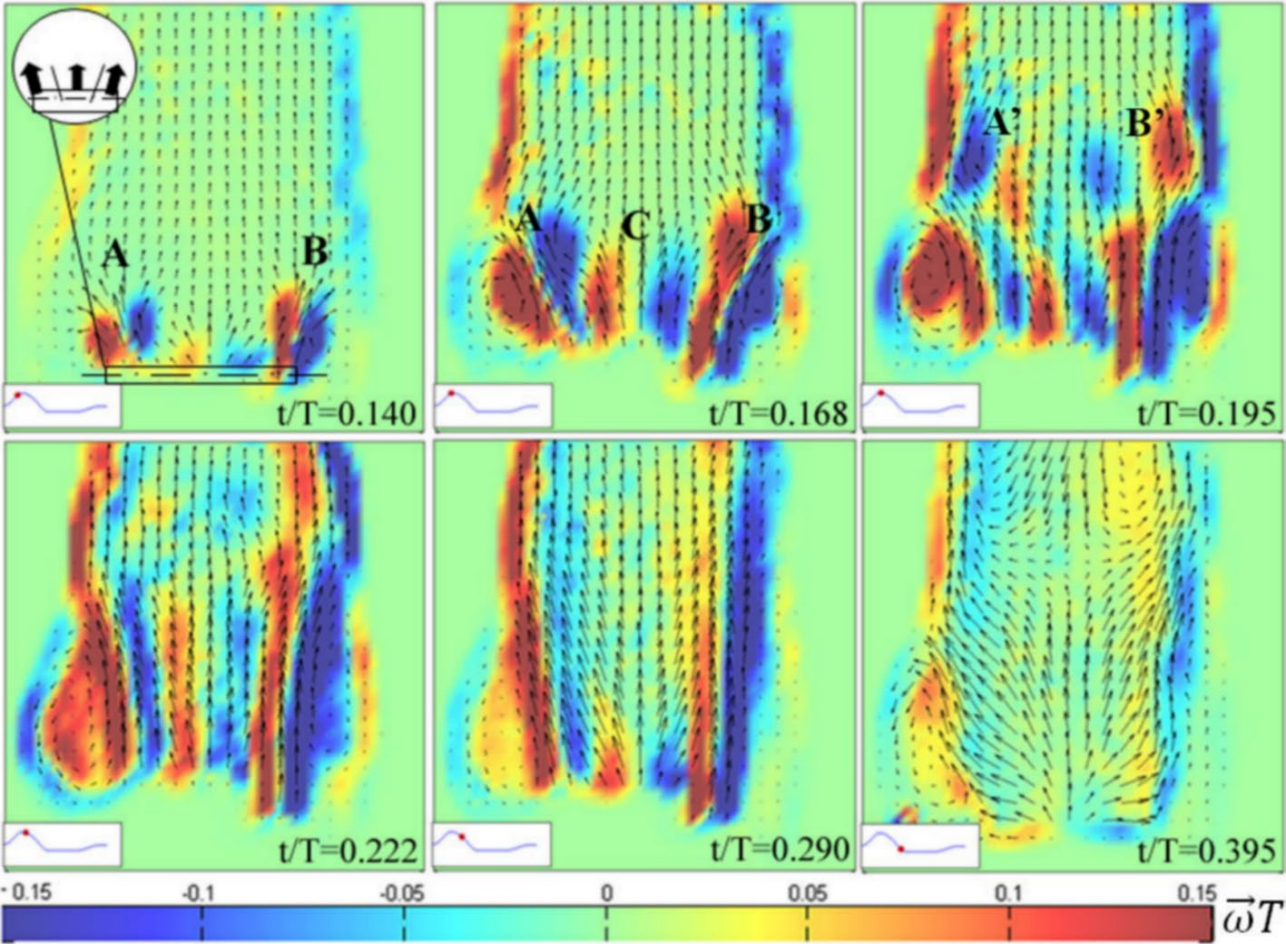
Phase averaged vector velocity field (*black arrows*) and non-dimensional vorticity 〈ωT〉 *color map* (*red* for counterclockwise vorticity and *blue* for clockwise vorticity) at different time instants (*red dots* on the flow rate curve) for the test case SV = 64 ml, T = 2.4 s. In particular, *A*, *B* and *C* are the three main jets formed downstream of the valve, *A′* and *B′* the evolution of *A* and *B* as the main eddies observed downstream the sinus

Figure 5 illustrates the phase averaged velocity field and the distribution of non-dimensional vorticity for six representative time instants (red dot on the reported aortic flow rate curve) during the ejection phase, for experiment 3. Shortly after the valve opening (t/T = 0.140) the triple jet pattern developing from the valve is clearly visible [9]. However, the two lateral jets (A and B for the left and right jet, respectively) are more intense than the central jet C, suggesting that the flow through lateral orifices starts to develop earlier than in the central region. Moreover, the jet emerging from the right leaflet (B) develops slightly earlier than the left one (A), according to the asymmetric phenomenon observed in the valve leaflets dynamics [62]. Such asymmetry should be related to the presence of the sinuses of Valsalva, as confirmed by the flow evolution at successive time instants [29]. At the peak of forward flow acceleration (t/T = 0.168) side jets A and B move upward to the aortic wall, farther B stretches up to the sinotubular junction more than jet A. A strong recirculating vortex generated by the left jet fills the sinuses of Valsalva, while only a smaller recirculation zone appears on the right side. The central jet is now of the same intensity of the side ones, but shortest. At t/T = 0.195 (peak systole) two structures (A′ and B′ in the vorticity map) separate from the two side jets and form a vortex ring that moves up leaving the investigated region (t/T = 0.222). At that instant, the vorticity layers in correspondence of the boundaries continue to move upwards, decreasing in intensity. During the deceleration phase (t/T = 0.290) a significant decreasing of the vorticity intensity is observed, in particular this is evident in correspondence of the sinuses of Valsalva. At the end of the systolic ejection (t/T = 0.395) the valve closure is marked by a flow inversion appearing in the upper part of the aortic root. Noteworthy, a flow asymmetry can still be appreciated, thus suggesting a possible asymmetry in the leaflets closing dynamics.

**Fig. 6:**
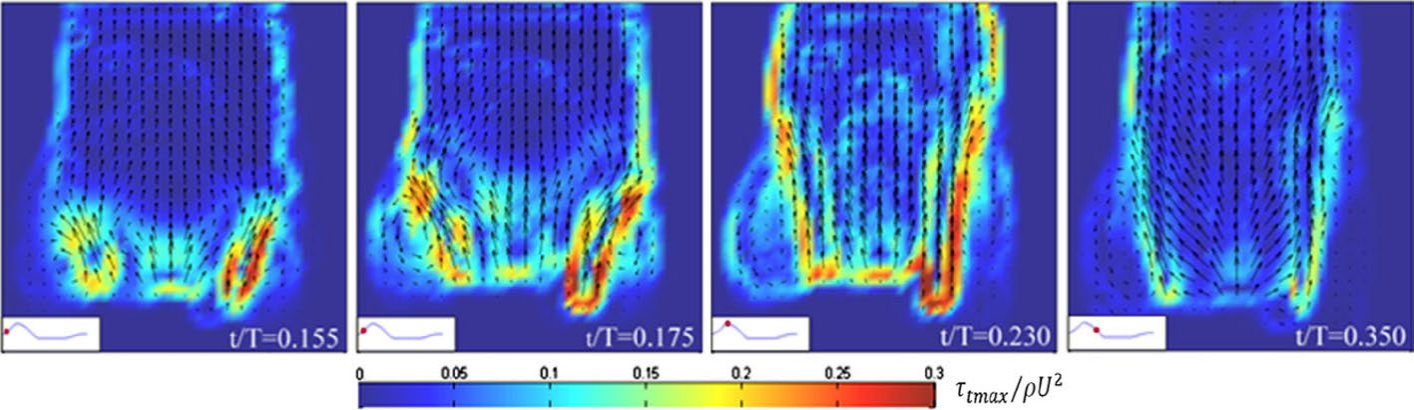
Phase averaged velocity field and non-dimensional maximum viscous shear stress τ_tmax_/ρU^2^ (*color map*) at different time instants for the test case SV = 64 ml, T = 2.4 s

Figure 6 shows the phase-averaged velocity field and the spatial distribution of the non-dimensional maximum viscous shear stress τ_tmax_/ρU^2^ at four time instants in the ejection phase, for the same experiment. The valve induces a complex texture of high shear layers, due to the development of the three jets. Both the distribution and the magnitude of τ_tmax_/ρU^2^ present a strong asymmetry with respect to the longitudinal axis, the region close to the right leaflet is indeed the mostly solicited. Again this asymmetry resembles the one observed in the valve dynamics. Results also show how regions characterized by higher values of maximum shear stress (i.e. τ_tmax_/ρU^2^ ≥ 0.2–0.25) are not confined in the region close to the valve. As time evolves, they rather tend to extend along the root boundary up to distances equal to more than twice the vessel diameter. Moreover, the residence time of τ_tmax_/ρU^2^ ≥ 0.2–0.25 is larger than two-thirds of the ejection period. Spatial distribution and temporal duration of maximum shear stress then give a preliminary, but fundamental, information about the potential damage on blood cells due to the action of the flowing fluid across the valve.

### Potential damage to blood particles

In biomedical devices, such as MHVs, shear stress distribution is usually quite far from the physiological condition both for spatial distribution and amplitude, thus demanding the quantification of shear-induced blood trauma to assess the safety and efficacy of the device prior to its marketing [1, 53].

**Fig. 7:**
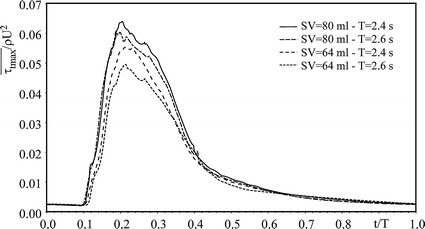
Non-dimensional maximum shear stress averaged over the aortic root area $\bar{\tau }_{tmax}$/ρU^2^ as a function of non-dimensional time t/T for different haemodynamic working conditions

Shear stress level and duration are recognized as primary factors driving blood trauma [54]. Hence we averaged the maximum shear stress over the investigated area to compare its overall behaviour during the whole cycle for different haemodynamic working conditions. To this aim we plotted the non-dimensional averaged stress $\overline{{\tau_{tmax} }}$/ρU^2^ as a function of t/T (Fig. 7). Results show that maximum of $\overline{{\tau_{tmax} }}$/ρU^2^ increase with both SV and T, the effect of T becoming smaller for larger SVs. Moreover, the area underlying the curves seems to depend on both SV and T, suggesting that blood cells damage due to mechanical stresses in time is possibly sensitive to bulk flow conditions. The above idea was explored by calculating a first estimation of red cells HI. In the power law here considered to evaluate HI, the exposure time t_exp_ was calculated as the time required to cross the investigated region with average velocity U while the ‘active’ shear stress τ was assumed equal to the maximum value of $\bar{\tau }_{tmax}$. The following values were recovered: HI = 0.0000284% for SV = 64 ml, T = 2.4 s; HI = 0.0000701% for SV = 80 ml, T = 2.4 s; HI = 0.0000205% for SV = 64 ml, T = 2.6 s; HI = 0.0000507% for SV = 80 ml, T = 2.6 s. Thus, HI was found to increase quite significantly with SV (with an estimated factor of about 2.5 from SV = 64 ml to SV = 80 ml) and to slightly decrease as T increases (with an estimated factor of about 0.7 from T = 2.4 s to T = 2.6 s). Interestingly, the computed values of HI are not far from previous studies and about one order of magnitude smaller than those estimated after one passage through the healthy blood system (HI = 0.00058%, value reported in [38]), suggesting the safety of the tested valve from the haemolysis point of view although a reliable estimation of blood trauma potential of mechanical valves is far from being a sufficiently clarified issue due to the limitations of a power-law approach and the scarcity of experimental data on RBCs in physiological flows. A specific study on this topic, based on the present results, is currently in progress.

## Conclusions

Global haemodynamic performance of a BMHV in aortic position was tested measuring simultaneously different metrics varying the hydrodynamic working conditions, allowing an all-around view of the valve behaviour. In particular, we considered transvalvular pressure drop and EOA, leaflets opening/closing angle, local velocity and shear stresses, potential damage of blood cells. Results allowed to appreciate the asynchronous behaviour of the two leaflets, possibly due to their different orientation with respect to the sinuses of Valsalva and to even minor differences in leaflets design. The local flow field analysis showed the presence of asymmetric fluid structures particularly evident in the shear stress distribution. The shear stress in the region close to the valve allowed a first estimate of the potential damage of red blood cells due to mechanical action; also variations in the HI were found as the bulk flow conditions were varied.

The benefit of an integrated approach stimulates some observations that can be made only analysing the results from a comprehensive perspective:

- both the EOA and the HI were found to be affected by bulk flow conditions; in particular, they both increase with SV and as T decreases, thus suggesting that the global and the local performance of the prosthesis show opposite trend with changes in the haemodynamic regime. In other words, the optimization of the overall prosthetic valve performan*ce results from the best possible compromise in the control of heart work overload and blood cells damage due to the valve itself. Moreover, also the leaflets dynamics was found to improve (in both leaflets synchronicity and maximum opening angle) with a SV increasing. We can hence speculate that flow dependence of the EOA (i.e. of the global performance of the prosthesis) actually is a consequence of the response of valve dynamics to flow changes. On the contrary the local performance, or at least the haemolytic potential, seems to appear more sensitive to flow intensity variations per se than to geometrical orifice area, although improved as a consequence of larger flow.
- A strong asymmetry in the shear stress distribution was observed. A relevant clinical implication can be seen in that result, which can possibly explain the asymmetric distribution of pannus/thrombotic deposits that is sometimes reported for explanted BMHV [38, 63]. Whether local flow dynamics asymmetry is related to the asynchronous behaviour of valve leaflets *and/or* vice versa, and the latter to valve implant orientation with respect to the sinuses of Valsalva, deserves further investigation. A promising approach might be seen in a combination of in vitro tests like those here presented and in silico tests able to predict blood particles trauma [10, 40].

## Availability of data and materials

The dataset supporting this study—in which results and discussion sections are based—are included within the article as additional files: one spreadsheet for the pressure fields across the valve (pressure_data.xls) and 8 for the velocity fields (2 for each of the 4 experiments performed, corresponding to the horizontal and vertical components) within the investigated domain.

As far as the pressures are concerned, the reported tests are labelled following Table 1. Each test columns report: the experiment time, the ventricular pressure, the aortic pressure, and the displacement acquired by the LVDT sensor placed at the bellow. Each acquisition is composed by 1200 samples.

As far as the velocity fields are concerned, the phase averaged velocity fields corresponding to the experiments:

- SV = 64 ml; T = 2.4 s T = 2.6 s (U_64_2_4.dat, V_64_2_4.dat; U_64_2_6.dat, V_64_2_6.dat)
- SV = 80 ml; T = 2.4 s T = 2.6 s (U_80_2_4.dat, V_80_2_4.dat; U_80_2_6.dat, V_80_2_6.dat) have included. Each file represent the time history of the corresponding velocity component: the number of rows corresponds to the size of the velocity field (50 × 51) while the number of columns corresponds to the number of acquired frames (1118 for experiments @T = 2.6 s, 1132 for experiments @T = 2.4 s).

## Additional files

**Additional file 1.** Pressure data.

**Additional file 2.** Evolution of fluid particle trajectories.

**Additional file 3.** Velocity data for the performed experiments.

## Abbreviations

MHV: mechanical heart valve
BMHV: bileaflet mechanical heart valve
EOA: effective orifice area
RBC: red blood cells
PD: pulse duplicator
AA: ascending aorta
LVOT: left ventricle outflow tract
SV: stroke volume
FT: feature tracking
HI: haemolysis index
